# Involvement of Neutrophils in Metastatic Evolution of Pancreatic Neuroendocrine Tumors

**DOI:** 10.3390/cancers13112771

**Published:** 2021-06-02

**Authors:** Véronique Debien, Guillaume Davidson, Philippe Baltzinger, Jean-Emmanuel Kurtz, François Séverac, Alessio Imperiale, Patrick Pessaux, Pietro Addeo, Philippe Bachellier, Xiaoping Su, Irwin Davidson, Marie-Pierrette Chenard, Bernard Goichot, Gabriel G. Malouf

**Affiliations:** 1 Department of Oncology, Institut de Cancérologie Strasbourg Europe, ICANS, 67200 Strasbourg, France; je.kurtz@icans.eu; 2 Department of Cancer and Functional Genomics, Institute of Genetics and Molecular and Cellular Biology, CNRS/INSERM/UNISTRA, 67400 Illkirch, France; davidsoy@igbmc.fr (G.D.); baltzinp@igbmc.fr (P.B.); irwin@igbmc.fr (I.D.); marie-pierrette.chenard@chru-strasbourg.fr (M.-P.C.); 3 Internal Medicine Department, Hôpitaux Universitaires de Strasbourg, 67000 Strasbourg, France; bernard.goichot@chru-strasbourg.fr; 4 Department of Public Health and Epidemiology, Hôpitaux Universitaires de Strasbourg, 67000 Strasbourg, France; francois.severac@chru-strasbourg.fr; 5 Department of Nuclear Medicine, Hôpitaux Universitaires de Strasbourg, 67000 Strasbourg, France; a.imperiale@icans.eu; 6 Department of Surgery, Nouvel Hôpital Civil, Hôpitaux Universitaires de Strasbourg, 67000 Strasbourg, France; patrick.pessaux@chru-strasbourg.fr; 7 Department of Hepato-pancreatic Surgery, Hôpital de Hautepierre, Hôpitaux Universitaires de Strasbourg, 67000 Strasbourg, France; pietrofrancesco.addeo@chru-strasbourg.fr (P.A.); philippe.bachellier@chru-strasbourg.fr (P.B.); 8 Department of Bioinformatics and Computational Biology, MD Anderson Cancer Center, Houston, TX 77230-1402, USA; XSu1@mdanderson.org; 9 Department of Pathology, Hôpitaux Universitaires de Strasbourg, 67000 Strasbourg, France

**Keywords:** pancreatic neuroendocrine tumors, neutrophils-to-lymphocyte ratio, tumor microenvironment, neutrophils, complement, innate immunity, transcriptome

## Abstract

**Simple Summary:**

The neutrophil-to-lymphocytes (NLR) reflects the systemic inflammation. Based on a cohort of 144 patients treated for localized or metastatic well-differentiated pancreatic neuroendocrine tumors (pNET), we identified the NLR ≥ 4 to be associated with worse overall survival. Using MCP-Counter on a publicly available pNET RNA-sequencing dataset, we inferred the tumor microenvironment composition of 83 primary pNET and 30 liver metastasis. The neutrophils scores were statistically higher in liver metastasis relative to primary pancreatic tumors (*p* = 0.005). Gene set enrichment analysis further revealed activation of complement pathway signature in liver metastasis. Through combination of the neutrophil and complement pathway genes, we found that pNET can be classified into two clusters: Neu-Comp1 and Neu-Comp2. Notably, the Neu-Compt1 cluster was enriched in neutrophils and complement pathway signatures and was associated with liver metastasis. These data offer new insights into the role of inflammatory factors in the metastatic progression of the pNET.

**Abstract:**

Well-differentiated pancreatic neuroendocrine tumors (pNET) have an unpredictable natural history. The identification of both blood and tumor immune features associated with patients’ outcomes remains limited. Herein, we evaluated the best prognostic value of the neutrophils-to-lymphocyte ratio (NLR) in a cohort of 144 pNETs. The NLR ≥ 4 was associated with worse overall survival in both univariate analysis (HR = 3.53, CI95% = 1.50–8.31, *p* = 0.004) and multivariate analysis (HR = 2.57, CI95% = 1.061–6.216, *p* = 0.036). The presence of synchronous liver metastasis was identified as a prognostic factor in multivariate analysis (HR = 3.35, CI95% = 1.411–7.973, *p* = 0.006). Interestingly, the absolute tumor-associated neutrophils count was higher in liver metastasis as compared to their paired primary tumor (*p* = 0.048). Deconvolution of immune cells from the transcriptome of 83 primary tumors and 30 liver metastases reveals enrichment for neutrophils in metastasis relative to primary tumors (*p* = 0.005), and this was associated with upregulation of the complement pathway (NES = 1.84, *p* < 0.0001). Combining neutrophils signature and complement pathway genes, unsupervised clustering identified two pNETs subgroups, namely Neu-Comp1 and Neu-Comp2. Characterized by neutrophils infiltration and activation of the complement pathway, Neu-Comp1 was highly enriched for metastatic liver samples as compared to Neu-Comp2 (*p* < 0.0001). These data suggest the possible link between liver metastasis, complement pathway activation, and neutrophils infiltration in well-differentiated pNET and open avenues for targeting complement pathways in these tumors.

## 1. Introduction

Pancreatic neuroendocrine tumors (pNET) are the second most frequent tumor arising in the pancreas after adenocarcinoma, accounting for 1–3% of all pancreatic tumors [1]. Their incidence has been increasing over the last few years [2]. According to the Surveillance, Epidemiology, and End Results (SEER) registry, the incidence has reached 0.8 new cases per 100,000 persons per year in 2012 as compared to <0.1/100,000 per year in 1973 [2]. pNET is a heterogeneous disease in which the grade is the most important prognostic factor [3]. The three-trial grading system is based on the evaluation of tumors’ proliferative potential estimated by the percentage of Ki67 positive cells or mitotic count [4]. Grade 3 carcinomas are known to have the worst outcome with five-year overall survival (OS) estimated at 13%, while having a five-year OS for grade 1 (G1) and grade 2 (G2) tumors are 80% and 67%, respectively [5]. However, among well-differentiated grade G1 and G2 tumors, there is a true tumor heterogeneity leading to a distinct natural history. Among known prognostic factors for pNET, Ki67 expression (which is in part related to tumor grade) and stage at diagnosis (lymph node involvement, as well as the burden of liver metastases) are the most frequently used [3,6]. Carcinological surgery is the cornerstone of therapy for localized disease (although the wait-and-see strategy is an option for small G1 pNET) [3]. However, surgery can also be curative in some patients with liver metastases [7]. Outcomes in localized, well-differentiated G1 and G2 pNET are generally favorable, although the recurrence rate varies between 12–25% in the literature [8,9]. Therefore, the identification of prognostic biomarkers is an unmet need in this population [10]. Among the explored biomarkers, systemic inflammation has been recognized as and represents a hallmark of cancer [11].

Various biological parameters reflect systemic inflammation such as elevated blood neutrophil-to-lymphocytes (NLR) ratio, which fosters tumor proliferation and metastasis via inhibition of apoptosis, promotion of angiogenesis, and DNA damage [11,12,13]. Thus, the tumor-associated neutrophils (TANs) were shown to interact with tumor cells. Depending on various extracellular stimulations (e.g., IFNg, TGF-B), TAN may present an “immunosuppressive switch” from antitumor N1 phenotype to pro-tumoral N2 phenotype [14]. Moreover, TAN activity is dependent on the tumor type and location within the tumor (intratumoral versus stromal) [15].

The NLR has been already described as a prognostic factor in different types of tumors [16,17]. High NLR was related to patients’ poor overall survival among various metastatic tumors [11,12]. For example, in colorectal cancer (surgically treated localized disease, as well as in the metastatic setting), NLR > 5 was associated with a worse outcome [16]. Several studies described NLR as a prognostic factor of relapse and survival in all-grade resected pNET patients [18,19,20,21,22,23]. However, different cut-offs have been identified among the reported cohorts. Notably, beyond its simple calculation, NLR might be a surrogate for the immune tumor microenvironment (TME), whereas the presence of tumor-associated immune cells is generally assessed by immunohistochemistry (IHC) [24].

With the advent of immune checkpoint inhibitors, efforts have been made to perform the immune classification of various cancers types in different organs [25]. Those efforts revealed striking associations between somatic mutations and TME composition and response to immune checkpoint inhibitors [26]. However, to the best of our knowledge, no comprehensive subtypes immune TME profiling has been reported to date.

In the present work, we analyzed the association between NLR, clinicopathological tumor features, and patients’ outcomes in a cohort of well-differentiated pNET identifying higher NLR and TANs as features of liver metastasis. Furthermore, we have inferred the distribution of immune cells from the transcriptome of 83 primary pNET and 30 liver metastasis revealing striking associations between neutrophils enrichment, complement activation, and liver metastasis.

## 2. Materials and Methods

### 2.1. Patient Population

We recorded pNET patients’ data from five oncology centers in the area of Alsace, France. Patients were identified from the tumor board database between 1 January 2008 and 1 January 2019, and variables of interest were extracted from medical files. This study was approved by the Institutional Ethics Committee (no. 7435) of Strasbourg University Hospital and conducted in accordance with the principles outlined in the Declaration of Helsinki. Inclusion criteria were as follows: confirmed diagnosis of well-differentiated G1 and G2 pNET (all stages) reviewed by pathologists from national expert board TENPATH, adult patients, availability of white blood count analysis before treatment (surgery or medical treatment). Recorded data included: gender, age, tumor stage, pathology findings (grade, TNM staging, functional or not), prior medical history including a context of multiple endocrine neoplasia (MEN1), blood count characteristics, treatment characteristics, and survival. The definition of OS is the time between the diagnosis until death from any causes or the last day of follow-up. TNM stage was determined from the American Joint Committee on Cancer (AJCC) 2017 classification (seventh edition) [27]. The tumor grade and differentiation were defined according to 2017 WHO classification of pNET [28].

### 2.2. NLR Calculation

The lymphocyte and neutrophil counts were obtained from the white blood cells count (WBC) performed as close as possible (less than 3 months) to diagnosis or prior to the surgery for patients undergoing surgery. NLR was calculated by dividing the neutrophils absolute count by lymphocyte absolute count. The predictive value for OS of NLR was defined using the receiver operating characteristic (ROC) curve.

### 2.3. Immunohistochemistry Neutrophils and Lymphocyte Assessment

Three paired tissue samples from primary and liver metastasis were available for immunohistochemistry staining for the evaluation of tumor-associated neutrophils and lymphocytes. Neutrophils staining with the CD66b antibody (BioLegend^®^, cat. no. 305102) was performed according to the manufacturer’s instructions. For this purpose, two FFPE tissue sections from primary pNETs and their corresponding liver metastasis were prepared, washed in pH = 7.4 PBS, and stained with 10µg/mL of diluted CD66b antibody. In addition, tumor-associated lymphocytes were stained with the CD3 antibody (ThemoFicher^®^, cat. no. RM-9107-S, clone SP7) according to the manufacturer’s instructions. The density of staining was evaluated by counting the stained cells by two independent investigators (M.P.C. and V.D.) in one field x400, with an average of 10 fields. Acute appendicitis was used as a positive control for all stains.

### 2.4. RNA Sequencing Data and Bioinformatic Analysis

Raw data for RNA sequencing (RNA-seq) of 83 primary pNETs and 30 liver metastases were downloaded from a publicly available dataset (GEO: GSE98894) [29]. Raw reads were aligned using STAR v2.5.3a with the “--quantMode Transcriptome SAM” argument and by providing the GFF file from ENSEMBL v75; the gene expression level was then calculated using RSEM v1.3.3. To infer the distribution of immune populations, we used the MCP-Counter v1.2.0. package for R software [30]. The TME deconvolution tool allows for estimating in an abundance of 10 cell populations including eight immune and stromal cells’ population (neutrophils, myeloid dendritic cells, monocytic cells, B lineage, NK cells, cytotoxic lymphocytes, CD8 T cells, T cells), and two stromal cells’ populations (endothelial cells and fibroblasts), based on their expression score. To identify subgroups, unsupervised clustering was performed according to the Z-score and visualized as a heatmap with the ‘pheatmap’ package v1.0.12. The number of clusters was chosen empirically following the obtained dendrograms. The MCP-counter scores for immune cells were compared between identified clusters. To estimate the tumor-associated neutrophils score, we used a mean value of the neutrophil score (defined by expression of *CXCR1*, *CXCR2*, *FCGR3B* genes). The complement pathway was defined by the expression of *SERPINC1*, *C4BPB*, *PLG*, *APOC1, C3*, *APOA4CP*, *F2*, *TFPI2*, and *ITIH* genes.

To assess the differential gene expression between primary pNETs and liver metastasis, we used the Wald test for differential expression proposed by Love et al. and implemented in the Bioconductor package DESeq2 version 1.16.1 [31]. Genes with a high Cook’s distance were filtered out and independent filtering based on the mean of normalized counts was performed. *p*-values were adjusted for multiple testing using the Benjamini and Hochberg method [32]. Gene Set Enrichment Analysis (GSEA) was done using the GSEA software v4.0.3 with the pre-ranked algorithm on log2 (fold-changes) estimated by DESeq2, using the human hallmark gene sets from Molecular Signatures Database (MSigD) v7.1 [33,34]. Gene sets with a False Discovery Rate (FDR) < 0.05 were considered as significantly differentially expressed.

### 2.5. Statistical Analysis

The receiver operating characteristics (ROC) curve and its area under the curve (AUC) were used to obtain the best cut-off value for NLR based on the overall survival population. Survival outcomes were determined by the Kaplan–Meier method and compared by the log-rank test. Univariate and multivariate analyses were performed using a logistic regression model. The Akaike Information Criterion was used to keep variables associated with survival in multivariate analysis. The Cox proportional model was used for OS univariate and multivariate analyses.

The unpaired Student’s *t*-test and Chi^2^ test were performed to identify differences between groups and associations between NLR and categorical variables. The paired Student’s *t*-test was performed to evaluate the statistical difference in paired samples. The Kruskal–Wallis *t*-test was performed to compare three and more groups among each other. The analyses were performed with R Studio Version 1.1.463 and GraphPad Prism version 5.0 a. The results were considered statistically significant if *p* < 0.05.

## 3. Results

### 3.1. Patients’ Characteristics

Overall, 187 patients were identified from the tumor board database. Out of those, 24 and 19 cases were excluded because of missing data and G3 grade, respectively. Hence, 144 well-differentiated G1 and G2 pNET patients’ data were available for analysis. Patients’ demographics and characteristics are displayed in Table 1. Median patient age was 56 years (range: 20–81 years), with a slight male predominance (57%). Among the total population, 80 patients (55.6%) had the symptomatic disease at diagnosis, with 28 (19.4%) of them displaying functional tumors. Only 10 (6.9%) patients had known MEN1 syndrome. At diagnosis, synchronous metastases were present in 42 (29.2%) of patients, and 50 (34.7%) patients had lymph node involvement. A total of 129 (89.6%) patients underwent surgical treatment, with R0 and R1 resection in 112 and 17 cases, respectively.

The association between NLR and patient’s characteristics is reported in Table S1.

### 3.2. Survival of Patients According to NLR Ratio

**Figure 1:**
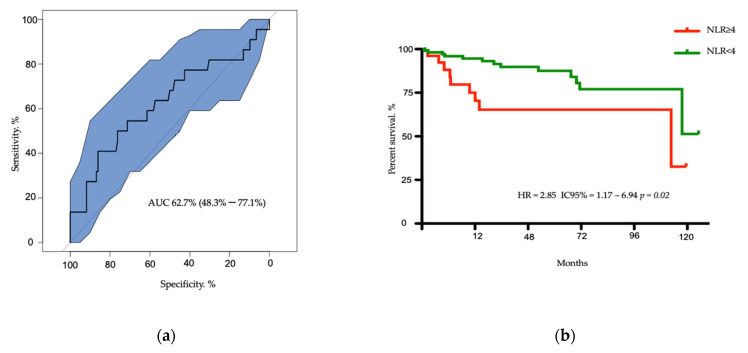
Determination of best cut off of neutrophil-to-lymphocyte ratio (NLR) and analysis of overall survival. (**a**) Receiver operating characteristic (ROC) curve for NLR to determine the best cut-off value: 4; AUC = 62.7) with a sensitivity of 41% and specificity of 86%. (**b**) Kaplan–Meier curves for patients overall survival according to NLR.

According to the AUC of 0.627, the best NLR cut-off was 4, with a sensitivity of 41% and a specificity of 86% (Figure 1a). Twenty-seven patients had an NLR ≥ 4. At the last time of follow-up, 13 patients died in the NLR < 4 subgroup as compared to 9 patients in the NLR ≥ 4 subgroup. With a median follow-up of 27 months, median OS was 113 months for patients with NLR ≥ 4 versus not reached (NR) for the subgroup of patients with NLR < 4 (HR = 2.850, CI 95% = 1.170–6.94, *p* = 0.02) (Figure 1b). The two-year OS rates were 74% and 96% in the NLR ≥ 4 and <4 subgroups, respectively.

### 3.3. Univariate and Multivariate Analysis for Overall Survival

In univariate analysis, the presence of metastasis (*p* = 0.006), lymph node involvement (*p* = 0.01) and NLR ≥ 4 (*p* = 0.004) were significantly associated with OS. Neither the continuous Ki67 value (*p* = 0.41) nor tumor T stage (*p* = 0.72) were identified as prognostic factors for OS (Table 2). In multivariate analysis, NLR ≥ 4 (HR = 2.57 CI = 1.061–6.216, *p* = 0.0036) and presence of synchronous liver metastasis (HR = 3.354 CI = 1.411–7.973, *p* < 0.006) were associated with poor OS (Table 2).

### 3.4. Association between NLR and Other Clinicopathological Features

**Figure 2:**
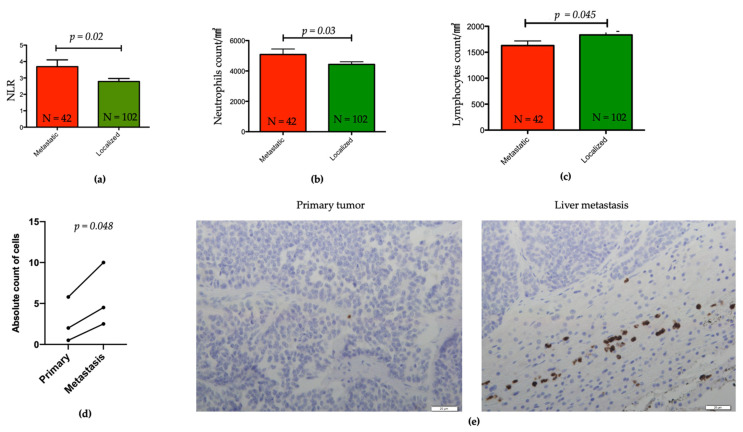
Distribution of patients’ blood neutrophils and lymphocytes counts in pNET. (**a**) Bar plot representing NLR from peripherical blood of patients with metastatic versus localized pNET; Student’s *t*-test: *p* = 0.02. (**b**,**c**) Bar plot of neutrophils and lymphocytes counts in patients with metastatic versus localized pNET; Student’s *t*-test: *p* = 0.03 and *p* = 0.045, respectively. (**d**) Dot plots representing the tumor-associated neutrophils counts in paired metastatic and in primary pNETs. (**e**) Representation of IHC CD66b staining of tumor-associated neutrophils in a primary pancreatic neuroendocrine tumor (left) and its corresponding liver metastasis (right), x400.

We then investigated the association between NLR ≥ 4 and other clinicopathological factors, such as age, gender, body mass index (BMI) <25 or ≥25kg/m^2^, presence of symptoms, Ki67 percentage, tumor size, tumor stage, lymph nodes, and distant metastasis (Table S1). Only the presence of synchronous metastasis was associated with increased NLR (HR = 2.32, CI = 0.98–5.51, *p* = 0.05). Notably, NLR was higher in metastatic as compared to localized pNET (*p* = 0.007, Figure 2a). This difference was associated with higher neutrophil counts and lower lymphocyte counts in metastatic relative to localized pNET subgroups (*p* = 0.03 and *p* = 0.045, respectively) (Figure 2b,c).

### 3.5. Evaluation of Tumors Associated Neutrophils in Liver Metastasis and Matched Primary pNETs

Out of our whole data set population, TANs were assessed by IHC in the matched primary and liver metastasis of three patients, for which material and informed written consents were available (Figure 2e). Interestingly, the absolute count of TANs was two-fold higher in the metastatic group (median range 2.5–10 neutrophils per field) compared to primary (median range 0.5–5.8 neutrophils per field) (*p* = 0.048) (Figure 2d), although the absolute level was low. The median value of CD3 lymphocytes per field in primary tumors was 7.9 and 5.5 in metastatic samples (Table S2)

### 3.6. Landscape of the Microenvironment Phenotypes in pNET

**Figure 3:**
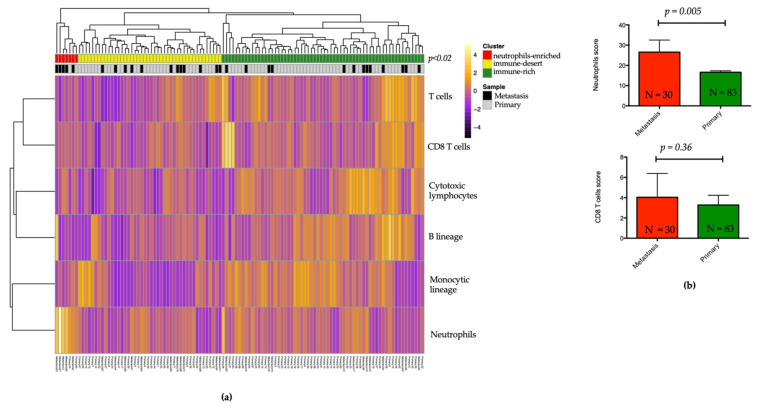
MCP-counter analysis of 113 pancreatic neuroendocrine tumors and distribution of immune cell scores in metastatic and primary tumors. (**a**) Heatmap showing unsupervised hierarchical clustering of immune cells in three clusters. (**b**) Box plot of neutrophils and CD8 lymphocytes score in liver metastasis versus primary pNET tumors.

To assess the tumor microenvironment (TME) composition in a large collection of 113 pNETs and explore the putative association with metastatic versus localized samples, we inferred the distribution of six immune populations (T cells, CD8 T cells, cytotoxic lymphocytes, B lineage, monocytic lineage, and neutrophils). Unsupervised hierarchical clustering using immune cell scores revealed three heterogeneous clusters: cluster 1 (*n* = 7; 6.2%), the “neutrophils-enriched”, with high enrichment for neutrophils (*p* < 0.0001); cluster 2 (*n* = 44; 38.9%), the “immune-desert”, with low immune cell infiltration; and cluster 3 (*n* = 62; 54.9%), the “immune-rich” cluster, with high T cells (*p* = 0.001) and cytotoxic lymphocytes (*p* < 0.0001) as compared to the other clusters (Figure 3a and Figure S1). Notably, the neutrophils-enriched cluster was tightly associated with metastatic samples (*n* = 5/7; 71.4%) relative to the remaining C2 (*n* = 12/44; 27.3%) and C3 (*n* = 13/62; 21%) clusters (*p* = 0.02).

### 3.7. Association between Neutrophils Infiltration, Complement Pathway Activation, and Metastatic Tumor Status

**Figure 4:**
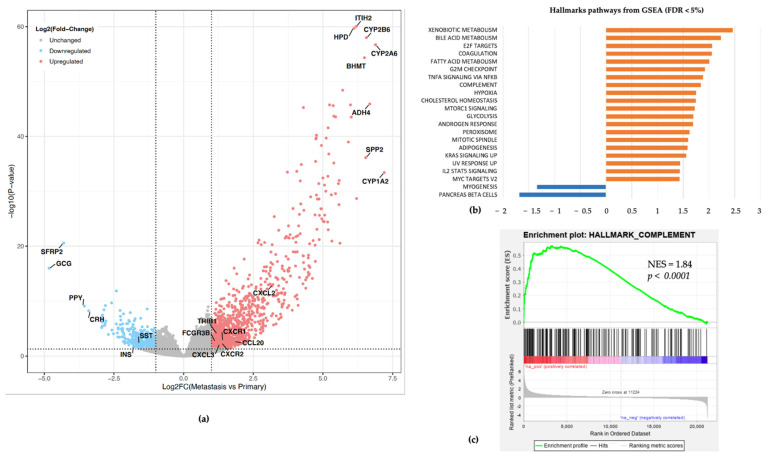
(**a**) Volcano plot of differential gene expression in liver metastasis versus primary pNET tumors. Each point represents a gene. Red represents upregulated genes, while blue is the downregulated one. (**b**) Bar plot representing differentially up- (orange) and down- (blue) regulated Hallmark pathways according to Gene Set Enrichment Analysis (GSEA analysis). NES: Normalized Enrichment Score. (**c**) Enrichment for complement pathway gene set in metastatic liver metastasis versus primary pNET tumors.

To further analyze if any specific molecular features are defining metastatic versus primary pNETs particularly regarding an immunosuppressive myeloid environment possibly linked with higher blood NLR, we compared the distribution of immune cells’ scores between primary and metastatic samples. Only the neutrophil score was higher in liver metastasis versus primary pNET samples (*p* = 0.005, Figure 3b). Conversely, no statistically significant difference was observed for CD8 lymphocytes (*p* = 0.36, Figure 3b). We further investigated differentially expressed genes between primary and metastatic tumors; overall, 1041 genes were overexpressed (FC ≥ 2; *p* < 0.05), and 341 genes were downregulated (FC ≤ −2; *p* < 0.05). Gene set enrichment analysis using the Hallmark set identified 25 gene sets with significant enrichment in metastatic relative to primary pNETs (FDR < 0.05) and only two gene sets downregulated (FDR < 0.05). Most upregulated gene sets included E2F targets, xenobiotic metabolism, fatty acid metabolism, G2M checkpoints, and hypoxia along with complement pathway (FDR < 0.05, *p* < 0.0001) (Figure 4).

### 3.8. Subtypes Classification of pNETs Using Neutrophils and Complement Pathway Signature

**Figure 5:**
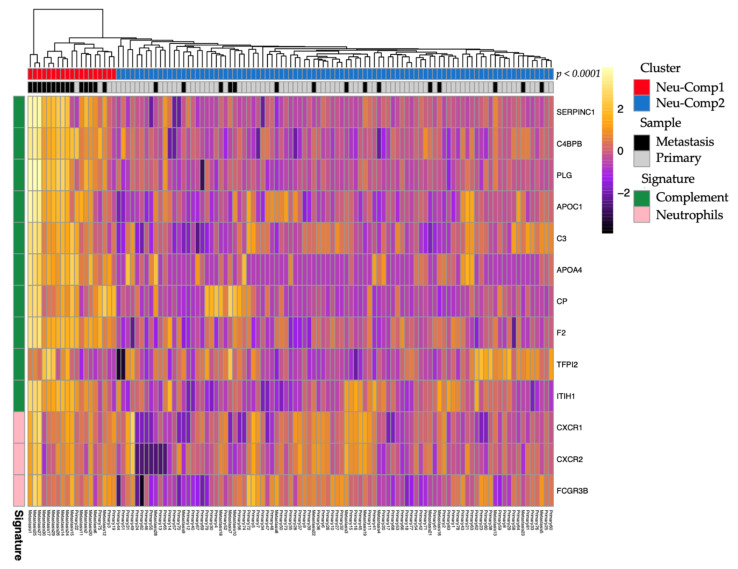
Unsupervised clustering of pNETs according to neutrophils and complement pathway gene expression signatures. Note that metastatic pNET cluster together as compared to localized pNETs (*p* < 0.0001).

Given the potential link between neutrophils and the complement pathway, we performed hierarchical clustering combining gene signatures of neutrophils and the top 10 expressed genes from the complement GSEA Hallmark gene sets. Unsupervised clustering identified two clusters. The first one (Neu-Comp1) (*n* = 19; 16.8%) was enriched for neutrophils and complement pathway as compared to the second one (Neu-Comp2) (*n* = 94; 83.2%). In addition, the Neu-Comp1 cluster was highly enriched for metastatic samples (*n* = 15; 78.9%) as compared to the Neu-Comp2 cluster (*n* = 19; 20.2%) (*p* < 0.0001, Figure 5).

## 4. Discussion

In this study, we identified NLR ≥4 as an independent biomarker for overall survival in well-differentiated pancreatic neuroendocrine tumors and found that it was associated with metastatic disease. We supposed that tumor-associated neutrophils could reflect the difference between primary and metastatic tumors. Moreover, our study investigating the immune TME in pNETs using transcriptome deconvolution, to our knowledge, the first of its kind, identifying TAN enrichment in liver metastasis relative to primary pNET. In addition, we unraveled an association between complement pathway activation and TAN enrichment that suggests the importance of the innate immune system in driving pNET metastasis.

Prognostic factors such as liver metastasis, tumor size, lymph node involvement, WHO grade classification, Ki67, or presence of symptoms have been previously described as prognostic biomarkers of recurrence-free survival [28,35,36,37]. In addition, scoring systems predicting integrating several clinicopathological parameters have been proposed by Genç et al. [6]. Recently, a high-risk, well-differentiated pNETs score was defined when two out of three of the following variables were present: tumor size > 20 mm, lymph node metastasis, and Ki67 > 5% or mitotic count > 2 [38]. Herein, we believe that NLR ≥ 4 might be added to those factors. To our knowledge, our study is the first to show elevated NLR in metastatic patients as compared to those with localized disease. Interestingly, the NLR ≥ 4 allowed us to identify a subgroup with a higher death risk in the first two years after diagnosis. To explain such heterogeneity, future investigations are needed to define the genetic and epigenetic molecular underpinnings of these tumors.

Few previous studies explored the role of NLR in well-differentiated pancreatic neuroendocrine tumors in European patients [6,39]. Other published series involved mainly resected patients in the Asian population [18,19,20,21,22,40,41]. Recently, a pooled analyses from RADIANT-3 and RADIANT-4 identified NLR < 2.58 to be associated with longer PFS in all subgroups, including pancreatic neuroendocrine tumors (*n* = 396, HR = 0.53 CI 95% 0.39–0.70) [42]. All patients enrolled in these trials had metastatic disease, and the majority of them have been already exposed to systemic treatments, including chemotherapy, which could have affected NLR. As NLR is an accessible biomarker of tumor-associated inflammation, we hypothesized that high NLR might be a surrogate marker of tumor microenvironment composition. To date, the correlation between circulating neutrophils and tumor-infiltrating neutrophils is inconsistent among solid tumors. For instance, TANs in pancreatic cancers were shown to be increased in patients with high NLR, although the correlation was not statistically significant [43]. At the functional level, neutrophils are involved in the anti-tumor activity (N1), as well as in the promotion of tumorigenesis (N2); thus, under the pressure of various cytokines they might participate in either angiogenesis and/or metastasis development [13,44,45].

To the best of our knowledge, the difference in TME composition between primary and metastatic pNET is poorly understood. Herein, by using the transcriptome deconvolution for the TME description, we have shown that neutrophils expression scores were higher in liver metastasis relative to primary pNETs, consistent with our IHC staining for available matched primary and liver metastasis. These data are keeping with results showing that higher infiltration of intratumoral neutrophils in localized well-differentiated pNETs has been associated with poor outcomes [46]. Thus, TANs might have an N2 pro-tumoral phenotype driving tumor aggressiveness. Further studies are needed to analyze the distribution and features of these cells using single-cell transcriptome sequencing.

To date, the role of immune cells in pNET has been investigated in several studies using IHC [24,47,48,49,50,51]. A higher level of tumor-infiltrating macrophages (TAMs) was shown to be associated with higher NLR. Both parameters have been statistically correlated with poor recurrence-free survival [22]. In another study, a high level of peritumoral TAMs was associated with lower disease-free survival [50].

Another interesting topic that is important to discuss in our study is the association between neutrophils infiltration with complement activation. Complement is a key factor in tissue inflammation, allowing cancer progression through the release of complement component 5a (C5a). Neutrophil stimulation by cytokines have been shown to activate the alternative complement pathway and release of C5 fragments, which further foster neutrophil proinflammatory responses [52,53]. This mechanism, possibly important for effective immune response, may play a key role in pNETs and highlight potential therapeutic targets to invigorate efficient immune response. Recently, Yang et al. reported compelling results about the potential involvement of the complement C1q activation in liver metastasis of patients with pancreatic adenocarcinomas [54]. Moreover, they showed that C1q is mainly expressed at tumor stroma rather in tumor cells and is involved in complement cascade. Mechanistic experiments further demonstrated that C1q would promote invasion and metastasis. These data are reminiscent with our observations in pNET, suggesting a role of complement pathway activation in hepatic metastasis along with high neutrophils infiltration.

Our study has several weaknesses. Firstly, the analysis of NLR was done retrospectively. The size of our cohort may present another limitation due to the rarity of the disease. However, to our knowledge, our study is one of the largest cohorts in the European population encompassing a large number of cases [20]. Secondly, the elevation of neutrophils or the decrease of lymphocytes may be the consequence of various physiological situations, like infection, or a result of systemic treatments like steroids [55]. Another significant limitation is the size of our setting for TAN evaluation. We only could perform IHC for a handful of paired samples. This limitation is mainly due to the rarity of the pathology, available tumor material, and retrospective nature of the study. Finally, another limitation is the lack of clinical annotation associated with the retrieved RNA-seq data that we analyzed.

Nonetheless, our study has several strengths. Firstly, it is a multicentric cohort on a period of more than 10 years with centralized cases reviewed by expert pathologists. Secondly, we have managed to establish that NLR ≥ 4, a value found in other solid tumors, is a prognostic tool for overall survival that is accessible for other clinicians and useful in practice. Thirdly, RNA-sequencing mining allowed us to deeply investigate the involvement of innate immunity in pNET. Our data suggest that the difference between localized and metastatic diseases may be related to the tumor microenvironment reflected by variance in NLR. Neutrophils infiltration in liver metastasis in our training and validation dataset strongly suggests that neutrophils may be involved in the development of metastasis, as it has been already reported for colorectal or breast cancer [56,57,58]. Finally, through the combination of complement pathways with neutrophils signature, we described two pNETs clusters separating metastatic from localized tumors. Altogether, we suggest that activation of the complement pathway may attract neutrophils, promoting not only the inflammation induced by cancer cells, but also their metastatic potential.

## 5. Conclusions

In summary, our work highlights the importance of tumor-related systemic inflammation biomarkers NLR and TNA as prognostic markers of metastasis in pNETs. Furthermore, this finding indicates the importance of complement activation along with neutrophils infiltration in metastatic pNETS, suggesting that targeting a complement pathway might open avenues for focusing on metastatic pNETs.

## Figures and Tables

**Table 1:** Baseline clinicopathological characterization of the current cohort.

Variable	*n* = 144
Median age, years (range)	56 (20–81)
Gender, male	82 (57%)
BMI, kg/m^2^	25,7 (16.5–46.3)
Missing data	19
Symptoms at diagnosis, Yes	80 (55.6%)
Functional tumor	28 (19.4%)
MEN1	10 (6.9%)
Size, median in mm, range	25 (5–120)
Missing data	9
Metastasis at diagnosis	42 (29.2%)
Ki67, median %	3 (1–20)
Grade 2 Grade 1	69 (48%) 75 (52%)
Size, T from AJCC 2017	
T1	58 (40.3%)
T2	33 (22.9%)
T3	48 (33.3%)
T4	4 (2.8%)
Lymph node status, N1	50 (34.7%)
Missing data	9
Surgery	129 (89.6%)
Surgical margins, R1	17 (11.8%)
Median NLR	2,31 (0.99–14.05)
Median neutrophils count,/mm^3^	4245 (1370–14470)
Median lymphocytes count,/mm^3^	1695 (420–4040)

NLR, neutrophil-to-lymphocyte ratio.

**Table 2:** Univariate and multivariate analysis of variables for overall survival.

*n* = 144		Univariate			Multivariate		
Variable	*n*	HR	CI 95%	*p* Value	HR	CI 95%	*p* Value
Age, >50 years	98	1.88	0.693–5.106	0.22			
Sex, male	82	2.46	0.908–6.676	0.08			
Ki67, continuous value	144	1.03	0.958–1.111	0.41			
Tumor size, T3-T4	52	1.17	0.494–2.783	0.72			
Lymph node involvement	50	3.27	1.315–8.117	0.01			
Metastasis	42	3.32	1.417–7.766	0.006	3.35	1.1411–7.973	0.006
NLR ≥ 4	27	3.53	1.502–8.313	0.004	2.57	1.061–6.216	0.036

NLR, neutrophil-to-lymphocyte ratio.

## Data Availability

The RNA-sequencing dataset is publicly available on GEO: GSE98894. Data is available upon reasonable request.

## Supplementary Materials

The following are available online at https://www.mdpi.com/article/10.3390/cancers13112771/s1, Figure S1: Bar plots of immune cells score in the three immune clusters: neutrophils-enriched (red), immune-desert (yellow), immune-rich (green). (a) T cells. (b) CD8. (c) Cytotoxic lymphocytes. (d) B lineage. (e) Monocytic lineage. (f) Neutrophils. Table S1: Association between neutrophil-to-lymphocyte ratio and clinical characteristics Table S2: Number of tumor-associated lymphocytes CD3 and neutrophils (CD66b) on three paired hepatic metastasis and primary well-differentiated pancreatic neuroendocrine tumor.
