# Supplementary material for: Involvement of Neutrophils in Metastatic Evolution of Pancreatic Neuroendocrine Tumors
Source: Cancers (Basel). 2021 Jun 2;13(11):2771. doi: 10.3390/cancers13112771 (PMC8199674; doi:10.3390/cancers13112771)
Supplement: Supplementary file 1 [file cancers-13-02771-s001.zip › cancers-1216751-supplementary.pdf]

Supplementary File

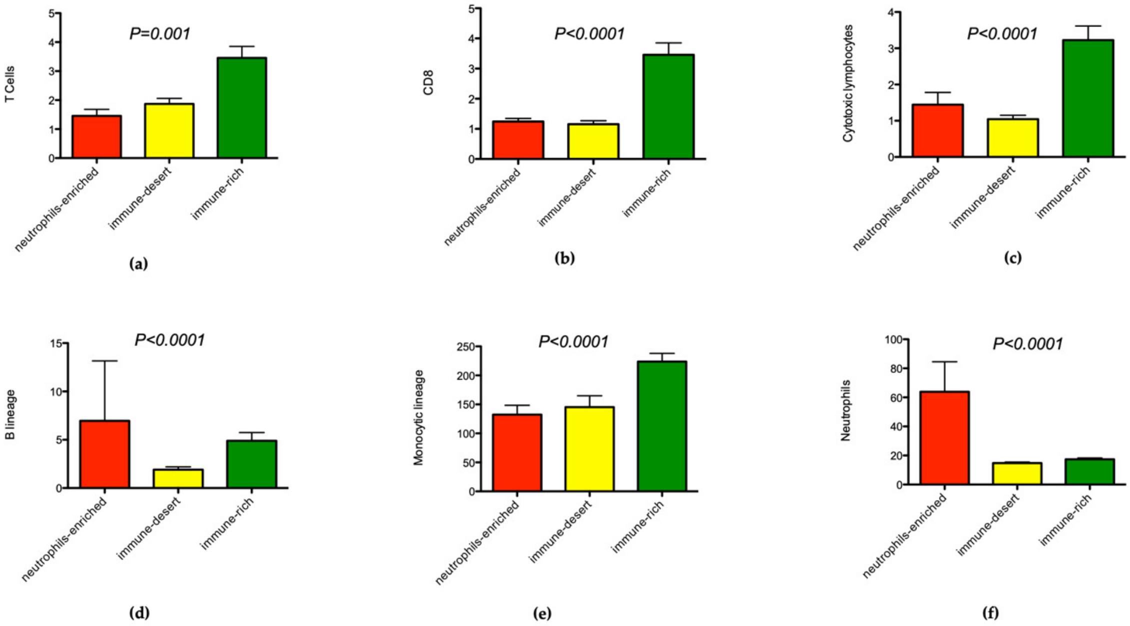

Figure S1. Bar plots of immune cells score in the three immune clusters: neutrophils-enriched (red), immune-desert (yellow), immune-rich (green). (a) T cells. (b) CD8. (c) Cytotoxic lymphocytes. (d) B lineage. (e) Monocytic lineage. (f) Neutrophils.

Table S1. Association between neutrophil-to-lymphocyte ratio and clinical characteristics

**Variables**	**N**	**NLR<4 N=117**	**NLR≥4 N=27**	**p-value**
**Age**	<50	34	10	0.88
	>50	62	17	
**Gender**	Male	64	18	0.26
	Female	53	9	
**BMI**	<25	47	6	0.22
	≥25	58	14	
	Missing Data	12	7	
**Symptoms**	No	51	13	0.67
	Yes	66	14	
**Size**	<30	59	13	0.70
	>30	50	13	
	Missing Data	8	1	
**Ki67, mean +/-SD**		5.0+/-4.9	5.2+/-4.6	0.84
**Ki67**	<5	70	12	0.14
	>5	47	15	
**Stage T, AJCC 2017**	T1/T2	73	18	0.71
	T3/T4	43	9	
**Lymph node involvement**	N0	70	15	0.37
	N1	38	12	
	Missing Data	9	0	
**Synchronous metastasis**	M0	87	15	0.05
	M1	30	12	

Table S2. Number of tumour-associated lymphocytes CD3 and neutrophils (CD66b) on three paired hepatic metastasis and primary well-differentiated pancreatic neuroendocrine tumour, the average of stained cells in 10 fields, x400.

	**CD66b**		**CD3**	
	Primary	Metastasis	Primary	Metastasis
**Patient 1**	2	4.25	16.7	10
**Patient 2**	5.8	10.4	5.6	3.1
**Patient 3**	0.5	2.5	1.5	3.3
**Mean**	2.8	5.7	7.9	5.5
